# Supplementary material for: Deep Transfer Learning for the Multilabel Classification of Chest X-ray Images
Source: Diagnostics (Basel). 2022 Jun 13;12(6):1457. doi: 10.3390/diagnostics12061457 (PMC9222116; doi:10.3390/diagnostics12061457)
Supplement: Supplementary file 1 [file diagnostics-12-01457-s001.zip › diagnostics-1764304-supplementary.pdf]

Deep Transfer Learning for the Multilabel Classification of Chest X-ray Images—Supplementary Material

Guan-Hua Huang [1,*], Qi-Jia Fu [1], Ming-Zhang Gu [1], Nan-Han Lu [2,3,4], Kuo-Ying Liu [5] and Tai-Been Chen [1,4]

[1] Institute of Statistics, National Yang Ming Chiao Tung University, Hsinchu 30010, Taiwan; qijia444@gmail.com (Q.-J.F.); eric956412@gmail.com (M.-Z.G.); ctb@isu.edu.tw (T.-B.C.)

[2] Department of Pharmacy, Tajen University, Pingtung City 90741, Taiwan; ed103911@edah.org.tw

[3] Department of Radiology, E-Da Hospital, I-Shou University, Kaohsiung City 82445, Taiwan

[4] Department of Medical Imaging and Radiological Science, I-Shou University, Kaohsiung City 82445, Taiwan

[5] Department of Radiology, E-Da Cancer Hospital, I-Shou University, Kaohsiung City 82445, Taiwan; ed102500@edah.org.tw

[*] Correspondence: ghuang@nycu.edu.tw; Tel.: +886-3-513-1334

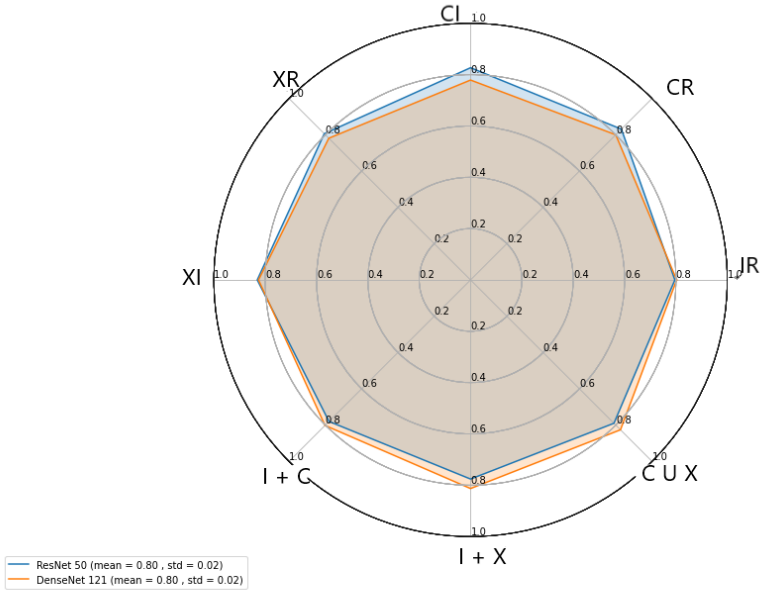

Figure S1. Radar plots for mean test AUCs from various transfer learning approaches. Abbreviations: IR = ImageNet pretraining with random initials, CR = ChestX-ray pretraining with random initials, CI = ChestX-ray pretraining with ImageNet pretraining initials, XR = CheXpert pretraining with random initials, XI = CheXpert pretraining with ImageNet pretraining initials, $\mathrm{I+C}$ = Concatenating ImageNet + ChestX-ray, $\mathrm{I+X}$ = Concatenating ImageNet + CheXpert, $\mathrm{C \cup X}$ = Cotraining ChestX-ray + CheXpert.

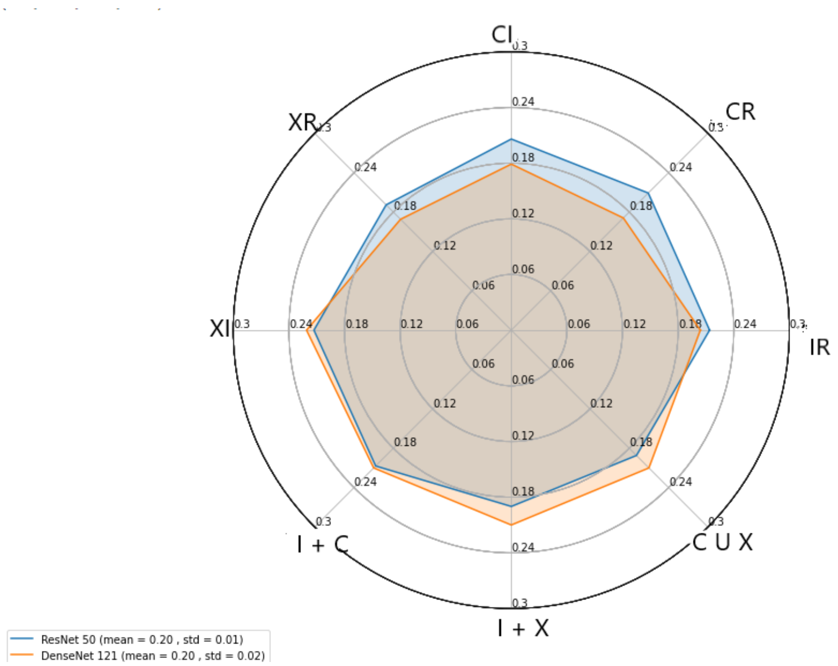

Figure S2. Radar plots for mean test APs from various transfer learning approaches. Abbreviations: IR = ImageNet pretraining with random initials, CR = ChestX-ray pretraining with random initials, CI = ChestX-ray pretraining with ImageNet pretraining initials, XR = CheXpert pretraining with random initials, XI = CheXpert pretraining with ImageNet pretraining initials, I + C = Concatenating ImageNet + ChestX-ray, I + X = Concatenating ImageNet + CheXpert, C ∪ X = Cotraining ChestX-ray + CheXpert.

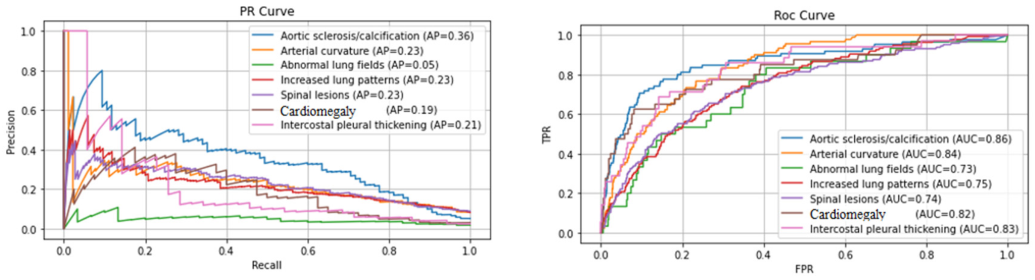

Figure S3. PR and ROC curves for each disease label in test data with initial weights from ImageNet pretraining with random initials in ResNet50.

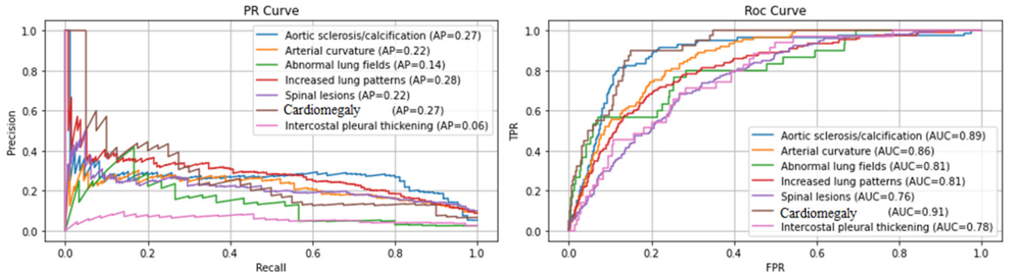

Figure S4. PR and ROC curves for each disease label in test data with initial weights from ChestX-ray pretraining with random initials in ResNet50.

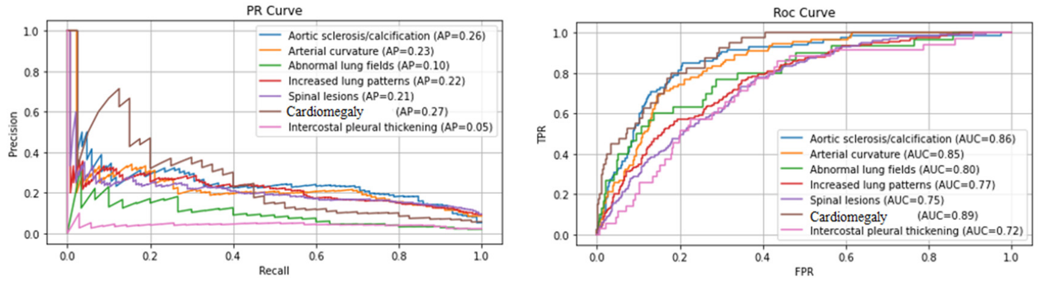

Figure S5. PR and ROC curves for each disease label in test data with initial weights from CheXpert pretraining with random initials in ResNet50.

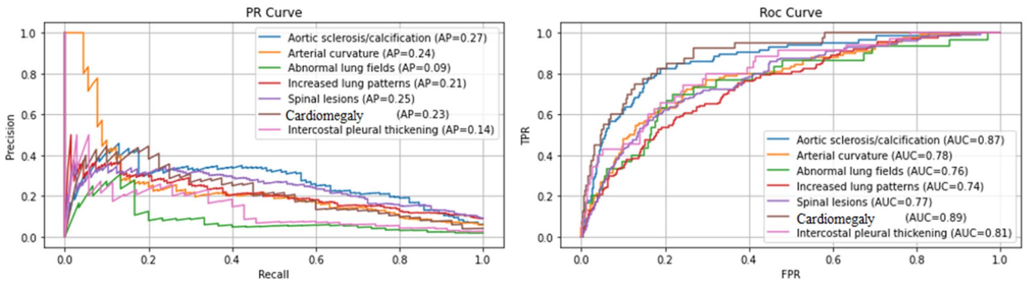

Figure S6. PR and ROC curves for each disease label in test data with initial weights from ImageNet pretraining with random initials in DenseNet121.

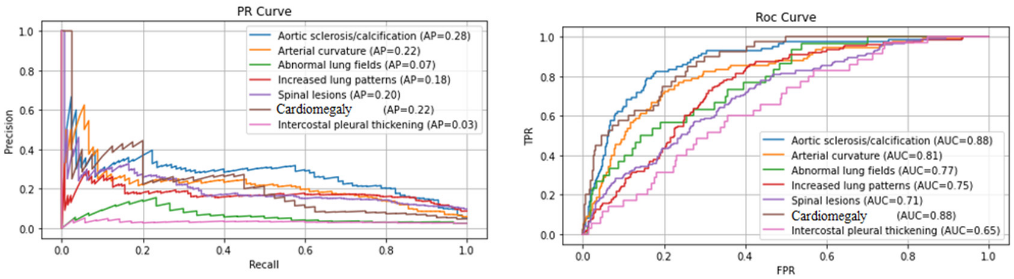

Figure S7. PR and ROC curves for each disease label in test data with initial weights from ChestX-ray pretraining with random initials in DenseNet121.

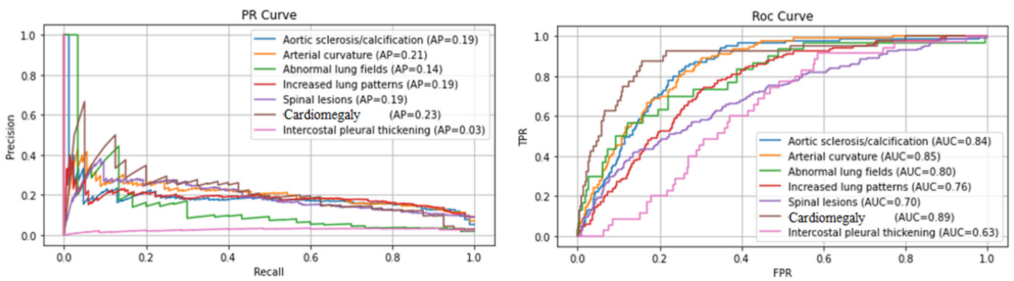

Figure S8. PR and ROC curves for each disease label in test data with initial weights from CheXpert pretraining with random initials in DenseNet121.

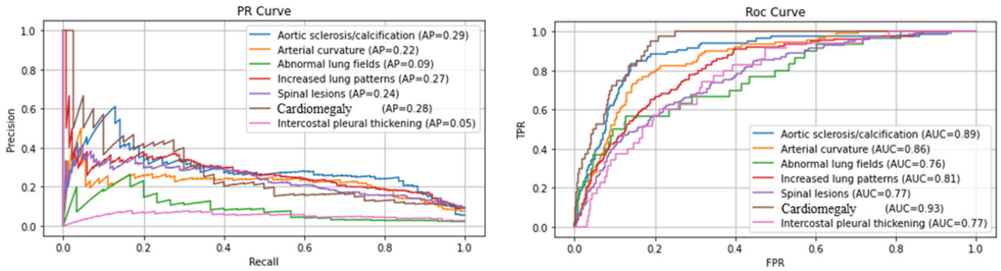

Figure S9. PR and ROC curves for each disease label in test data for initiating transfer learning with pretrained weights from ChestX-ray adopting ImageNet initials in ResNet50.

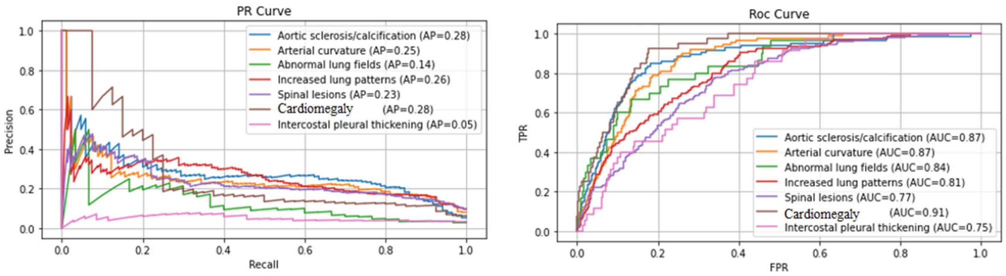

Figure S10. PR and ROC curves for each disease label in test data for initiating transfer learning with pretrained weights from CheXpert adopting ImageNet initials in ResNet50.

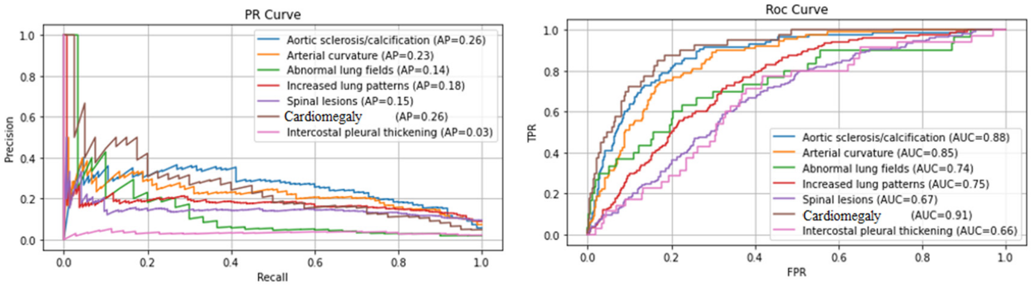

Figure S11. PR and ROC curves for each disease label in test data for initiating transfer learning with pretrained weights from ChestX-ray adopting ImageNet initials in DenseNet121.

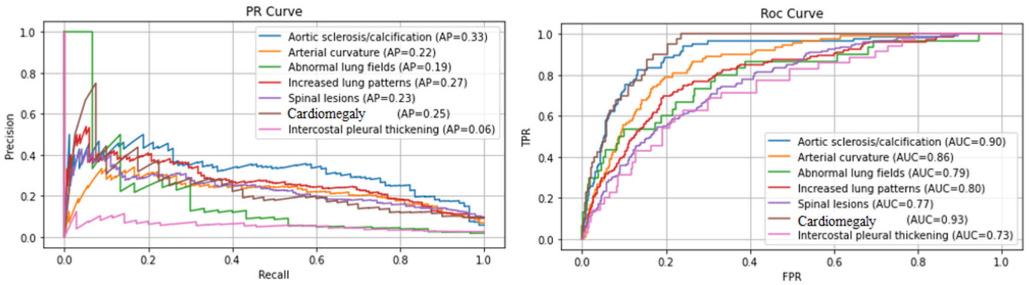

Figure S12. PR and ROC curves for each disease label in test data for initiating transfer learning with pretrained weights from CheXpert adopting ImageNet initials in DenseNet121.

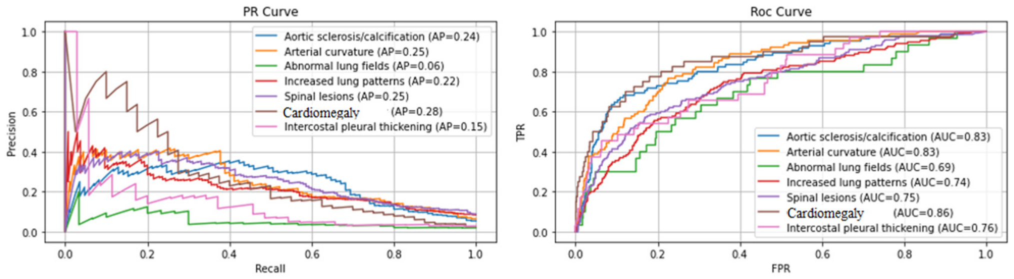

Figure S13. PR and ROC curves for each disease label in test data for concatenating transfer learning of ImageNet + ChestX-ray in ResNet50.

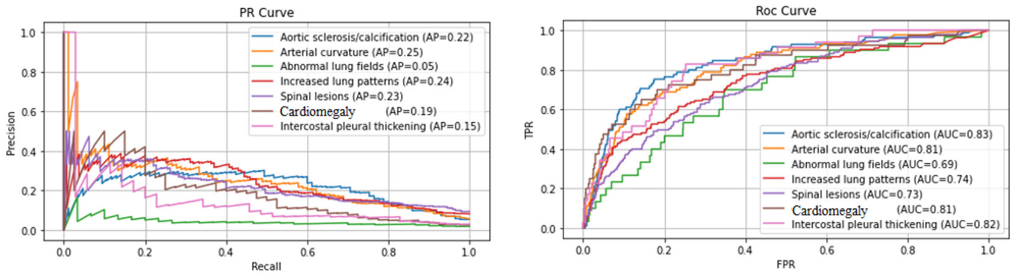

Figure S14. PR and ROC curves for each disease label in test data for concatenating transfer learning of ImageNet + CheXpert in ResNet50.

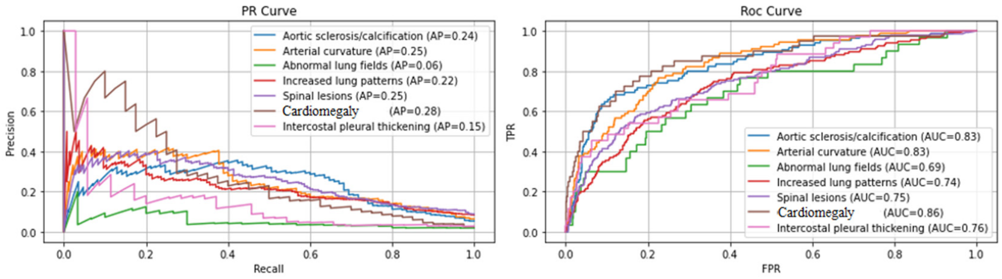

Figure S15. PR and ROC curves for each disease label in test data for concatenating transfer learning of ImageNet + ChestX-ray in DenseNet121.

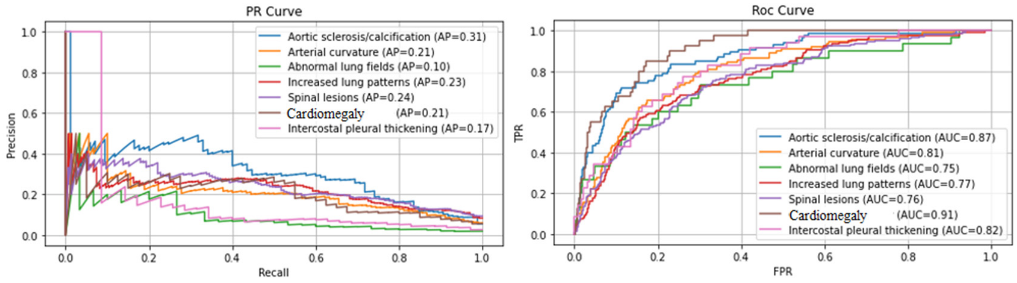

Figure S16. PR and ROC curves for each disease label in test data for concatenating transfer learning of ImageNet + CheXpert in DenseNet121.

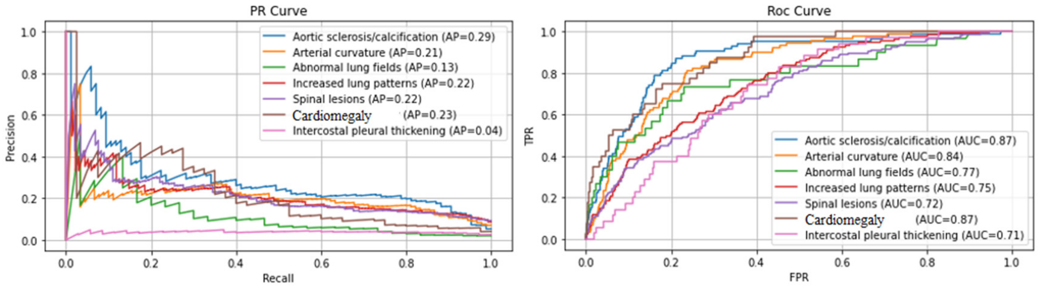

Figure S17. PR and ROC curves for each disease label in test data for co-training transfer learning of ChestX-ray + CheXpert in ResNet50.

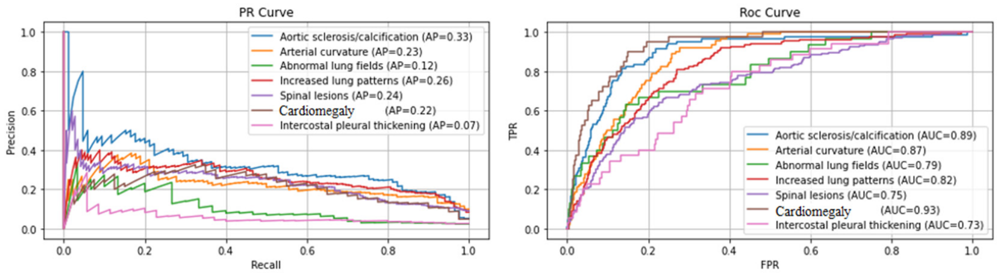

Figure S18. PR and ROC curves for each disease label in test data for co-training transfer learning of ChestX-ray + CheXpert in DenseNet121.
